# Characterizing genetic and antigenic divergence from vaccine strain of influenza A and B viruses circulating in Thailand, 2017–2020

**DOI:** 10.1038/s41598-020-80895-w

**Published:** 2021-01-12

**Authors:** Nungruthai Suntronwong, Sirapa Klinfueng, Sumeth Korkong, Preeyaporn Vichaiwattana, Thanunrat Thongmee, Sompong Vongpunsawad, Yong Poovorawan

**Affiliations:** Center of Excellence in Clinical Virology, Faculty of Medicine, Chulalongkorn University, Bangkok, 10330 Thailand

**Keywords:** Evolution, Genetics, Microbiology, Molecular biology

## Abstract

We monitored the circulating strains and genetic variation among seasonal influenza A and B viruses in Thailand between July 2017 and March 2020. The hemagglutinin gene was amplified and sequenced. We identified amino acid (AA) changes and computed antigenic relatedness using the *P*_epitope_ model. Phylogenetic analyses revealed multiple clades/subclades of influenza A(H1N1)pdm09 and A(H3N2) were circulating simultaneously and evolved away from their vaccine strain, but not the influenza B virus. The predominant circulating strains of A(H1N1)pdm09 belonged to 6B.1A1 (2017–2018) and 6B.1A5 (2019–2020) with additional AA substitutions. Clade 3C.2a1b and 3C.2a2 viruses co-circulated in A(H3N2) and clade 3C.3a virus was found in 2020. The B/Victoria-like lineage predominated since 2019 with an additional three AA deletions. Antigenic drift was dominantly facilitated at epitopes Sa and Sb of A(H1N1)pdm09, epitopes A, B, D and E of A(H3N2), and the 120 loop and 190 helix of influenza B virus. Moderate computed antigenic relatedness was observed in A(H1N1)pdm09. The computed antigenic relatedness of A(H3N2) indicated a significant decline in 2019 (9.17%) and 2020 (− 18.94%) whereas the circulating influenza B virus was antigenically similar (94.81%) with its vaccine strain. Our findings offer insights into the genetic divergence from vaccine strains, which could aid vaccine updating.

## Introduction

Influenza A and B virus infection remains a common cause of respiratory disease worldwide^1^. The majority of seasonal influenza viruses in circulation are influenza A(H1N1)pdm09, A(H3N2), and two lineages of the influenza B virus (B/Yamagata-like lineage and B/Victoria-like lineage)^2^. Annual influenza vaccination can reduce the risk of morbidity and mortality in infected individuals, but in some years the influenza vaccine offers lower than expected efficacy due to antigenic differences against the strains circulating in the community^3^. In 2014, influenza A(H3N2) clades 3C.2a and 3C.3a have emerged and contributed to reduce the vaccine effectiveness (VE). Reductions in the observed vaccine effectiveness may result from virus evolution over the period and the nature of the egg adaptive substitutions acquired in the vaccine^4–6^. Although influenza A(H1N1)pdm09 evolved at a slower rate than A(H3N2), newly emerging genetic groups have been reported^7^. In addition, B/Victoria-like lineage strains with deletions are also increasingly prevalent^8^.

The hemagglutinin (HA) protein is an important surface glycoprotein, which possesses the antigenic and receptor-binding sites (RBSs) and elicits the main neutralizing response^9,10^. The HA protein is divided into the globular head domain (HA1) and the stem domain (HA2). There are proposed antigenic sites on the HA1 of A(H1N1)pdm09 (Sa, Sb, Ca1, Ca2, and Cb) and on the HA1 of A(H3N2) (A through E)^9,11,12^. Meanwhile, there are four major HA antigenic sites for influenza B virus (120 loop, 150 loop, 160 loop, and 190 helix)^13^. Accumulation of mutations in the HA protein, particularly on the antigenic sites, RBSs and the surrounding region, has enabled the continuous evolution and the emergence of new influenza virus strains^12,14,15^, which escape the existing neutralizing antibodies^16^. As a result, the influenza virus strains to be included in the annual vaccine composition are carefully evaluated each year^8,17^.

Although influenza activity in Thailand occurs throughout the year, it is often bimodal^18^. Most influenza infection occur in the rainy season (August to November), but frequent infection can also occur in the cooler and drier months of January to March^18,19^. Thailand is geographically located in the northern hemisphere, but the vaccine formulation for the southern hemisphere is used. Thai people are annually vaccinated between April and May which corresponds to the beginning of the peak of influenza activity^20^. Typically, the vaccine composition for the southern hemisphere is determined many months before the actual influenza season begins in order to allow time for production and distribution^21,22^. Occasionally, the selected strains to be included in the vaccine are not well-matched (antigenically dissimilar) with the actual circulating strains and subsequently resulted in the poor vaccine effectiveness (VE) as happened during the 2014–2015^23,24^.

Periodic monitoring of the influenza virus strains circulating in the region is important for antigenic characterization and improved vaccine design. Here, we examined the influenza incidence from July 2017 and March 2020 from samples submitted for routine diagnostics from three hospitals in Thailand. We characterized the genetic and antigenic variations of the HA gene on 90 strains of influenza A(H1N1)pdm09, 90 strains of influenza A(H3N2), and 81 strains of influenza B virus sampled monthly over the study period. Deduced amino acid changes were mapped on the HA three-dimensional structures, and the computed antigenic relatedness was analyzed for each influenza (sub)types.

## Materials and methods

### Sample collection and ethics statement

Respiratory specimens comprising of nasopharyngeal swabs were submitted for testing as part of the routine influenza surveillance from July 2017 to March 2020 from patients with influenza-like illness (ILI). ILI was defined as fever (body temperature > 38 °C) combined with respiratory symptoms (cough, nasal congestion, runny nose, sore throat). A total of 17,480 samples from Bangpakok 9 International Hospital (n = 14,743), King Chulalongkorn Memorial Hospital (n = 266), and Chum Phae Hospital (n = 2,471) were tested. Viral RNA was extracted directly from clinical samples by using Ribospin vRD II according to the manufacturer’s instructions (GeneAll Biotechnology, Seoul, Korea). Real-time reverse-transcription quantitative polymerase chain reaction (RT-qPCR) was used to identify A(H1N1pdm09), A(H3N2), and the influenza B virus as previously described^25^. Laboratory-confirmed influenza B-positive samples were subjected to cDNA synthesis with primer FluB (5′-AGCAGAAGCA-3′) and ImProm-II Reverse Transcription System (Promega, Madison, WI, USA), followed by multiplex PCR and melting-curve analysis^26^. The study protocols were approved by the Institutional Review Board of Faculty of Medicine of Chulalongkorn University (IRB No. 127/61). Informed consent was obtained from all subjects, and all methods were carried out in accordance with relevant guidelines and regulations.

### Amplification of the hemagglutinin gene

Samples positive for influenza A(H1N1)pdm09 (n = 90), influenza A(H3N2) (n = 90), or influenza B virus (n = 81) sampled monthly throughout the study period were subjected to conventional RT-PCR to amplify the entire HA gene and Sanger sequencing. Influenza A(H1N1)pdm09 and A(H3N2) were subjected to cDNA synthesis using primer Uni12 (5′-AGCAAAAGCAGG-3′). The primer sets for both influenza A and B viruses have been described elsewhere^27,28^. PCR mixture of 25 μL contained 5 μL of AccuStart II GelTrack PCR SuperMix (Quantabio, Beverly, MA, USA), 0.25 mM MgCl_2_, 0.5 μM each of forward and reverse primers, and 3 μL of cDNA template. Amplification conditions were 94 °C for 3 min, 40 cycles of 30 s at 94 °C, 30 s at 55 °C, 90 s at 72 °C, followed by 7 min of final extension at 72 °C. Amplicons were agarose gel-purified and subjected to Sanger sequencing. Nucleotide sequences were assembled using SeqMan Pro (DNASTAR, Madison, WI, USA) and deposited in the GenBank database under the accession numbers MT803149–MT803238 for A(H1N1)pdm09, MT803239–MT803328 for A(H3N2) and MT803397–MT803477 for influenza B viruses (Table S1).

### Genetic characterization

Phylogenetic trees for each influenza virus were generated using the nucleotide sequences obtained from this study and additional HA sequences from other Thai strains previously identified and available from the National Center for Biotechnology Information (www.ncbi.nlm.nih.gov) and the Global Initiative for Sharing All Influenza Data (GISAID) (http://platform.gisaid.org) database. A total of 183 nucleotide sequences of influenza A(H1N1)pdm09, 232 nucleotide sequences of influenza A(H3N2), and 177 nucleotide sequences of the influenza B virus were aligned with the reference and vaccine strains for each influenza virus using MUSCLE. Nucleotide substitution models for the HA gene of influenza A(H1N1)pdm09 (TN93 + G), influenza A(H3N2) (HKY + G + I) and influenza B (HKY + G) were implemented in MEGAX^29^. Trees were constructed using the maximum-likelihood method and bootstrapping of 1,000 replicates. Deduced amino acid sequences were compared to the reference and vaccine strains to identify substitutions, which were noted at each branch node on the phylogenetic trees. Potential N-linked glycosylation sites on the HA gene was analyzed using the NetNGlyc 1.0 server^30^ and a threshold value of > 0.5.

### Visualization of amino acid residue changes on the HA structure

Publicly available three-dimensional crystal structures of influenza virus HA of influenza virus strains A(H1N1)pdm09 (A/California/04/2009, Protein Data Bank accession number 3LZG), A(H3N2) (A/Victoria/361/2011, accession number 4WE9), B/Victoria (B/Brisbane/60/2008, accession number 4FQM) and B/Yamagata (B/Yamanashi/166/98, accession number 4M44) were used to illustrate approximate residue position and changes on the HA. Identification of residues in the structure was performed using PyMOL Molecular Graphics System v1.3 (Schrödinger; https://www.schrodinger.com). Amino acid single-letter code preceding the numerical position represents residue in the vaccine strains, while amino acid single-letter code following the numerical position represents that found in this study. Vaccine strains were A(H1N1)pdm09 (A/Michigan/45/2015), A(H3N2) clade 3C.2a (A/Hong Kong/4801/2014), A(H3N2) clade 3C.3a (A/Switzerland/9715293/2013), B/Victoria-like lineage (B/Brisbane/60/2008), and B/Yamagata-like lineage (B/Phuket/3073/2013).

### Determination of selection pressure

The ratio of non-synonymous/synonymous substitutions (d*N*/d*S*) was considered when evaluating codon under selective pressure. d*N*/d*S* was analyzed using the mixed-effects model of evolution (MEME) and the fixed effects likelihood (FEL) methods. Both algorithms were in the HYPHY software implemented in the Datamonkey webserver (https://www.datamonkey.org/) ^31^. Positively selected residue was considered significant at P = 0.1.

### Estimation of computed antigenic relatedness

The antigenic relatedness of influenza virus was computed using the *P*_epitope_ model, which took into account the distinct antigenic sites between circulating strains and the vaccine strain by considering the epitope sites. The *P*_epitope_ model was calculated by the fraction of number of amino-acid substitutions in the dominant epitope and the total number of amino acids in that dominant epitope. The association between *P*_epitope_ model and antigenic distance measured by hemagglutinin inhibition assay or antigenic relatedness (efficacy, E) was determined by a mathematical formula. For A(H1N1)pdm09, E =  − 1.19 × *P*_epitope_ + 0.53 in which efficacy is 53% when the *P*_epitope_ = 0^32^. For A(H3N2), the association between the antigenic relatedness and *P*_epitope_ is given by E =  − 2.47 × *P*_epitope_ + 0.47 in which efficacy is 47% when *P*_epitope_ = 0^33^. For the influenza B virus, E =  − 0.864 × *P*_epitope_ + 0.6824 in which efficacy is 68.24% when *P*_epitope_ = 0^34^. The trend of computed antigenic relatedness was identified using R v3.6.0 (R Foundation for Statistical Computing, Vienna, Austria; https://www.r-project.org). The difference among annually computed antigenic relatedness was calculated using one-way ANOVA (P < 0.05 was considered statistically significant). Statistical analysis was done using Prism 8.0 (GraphPad, San Diego, CA, USA; https://www.graphpad.com).

## Results

**Figure 1:**
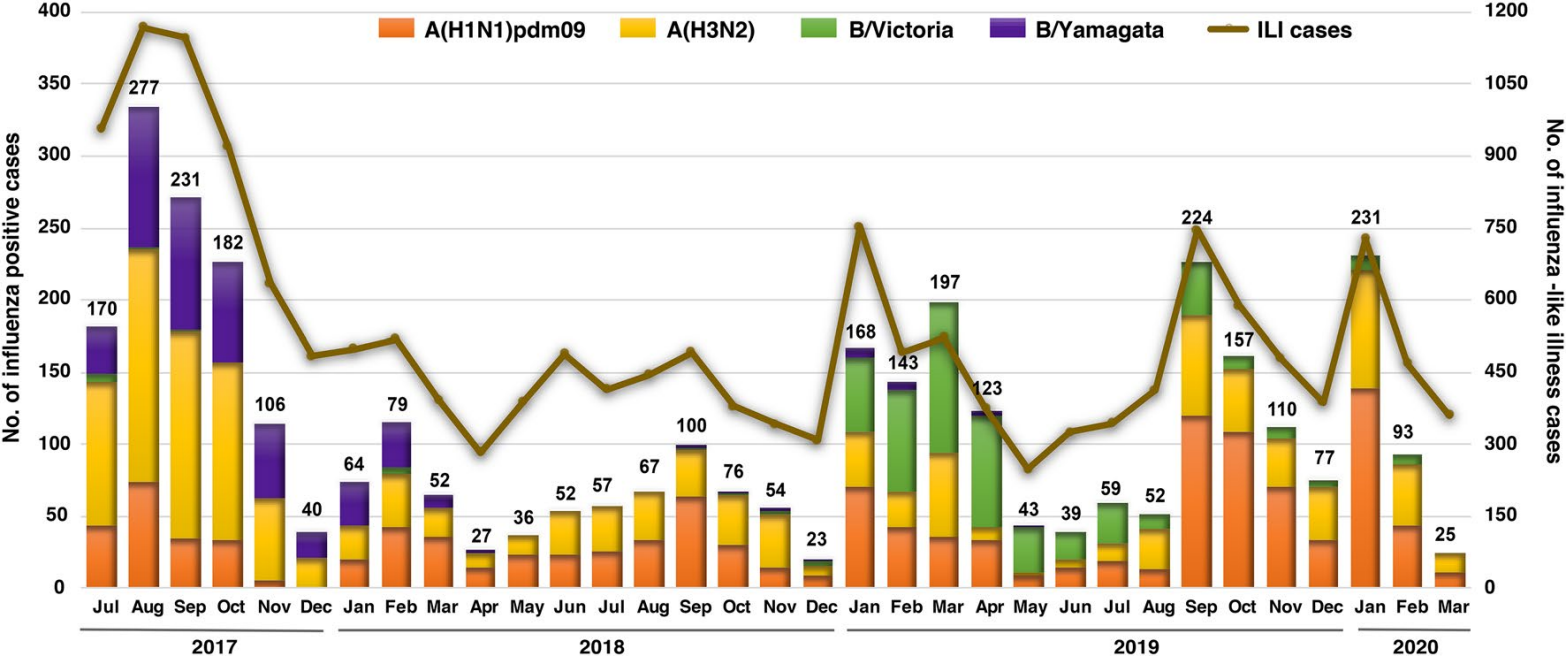
Monthly distribution of influenza A and B viruses from July 2017 to March 2020 (n = 17,480). Numbers above the bar graph indicate the monthly number of influenza virus-positive samples (left Y-axis) with respect to the monthly ILI samples (right Y-axis). The bar graph was generated by using Microsoft Excel (Office 365).

In this study, 20.90% (3654/17,480) of the nasopharyngeal swabs from ILI patients tested positive for influenza virus, of which 74.2% (2711/3654) were influenza A virus and 25.8% (943/3654) were influenza B virus. Typical monthly influenza activity peaked between August and November, and again in January to March (Fig. 1). Among influenza A virus-positive samples, 52.2% (1415/2711) were A(H3N2) and 47.8% (1296/2711) were A(H1N1pdm09). Influenza B virus of the B/Yamagata lineage predominated during the 2017–2018 season and accounted for 48.4% (456/943) over the study period. In comparison, influenza B/Victoria accounted for 51.6% (487/943) and was most predominant in the 2019–2020 season.

### Characterization of the clades/subclades based on HA sequences and their divergence from the vaccine strain

#### A(H1N1)pdm09

**Figure 2:**
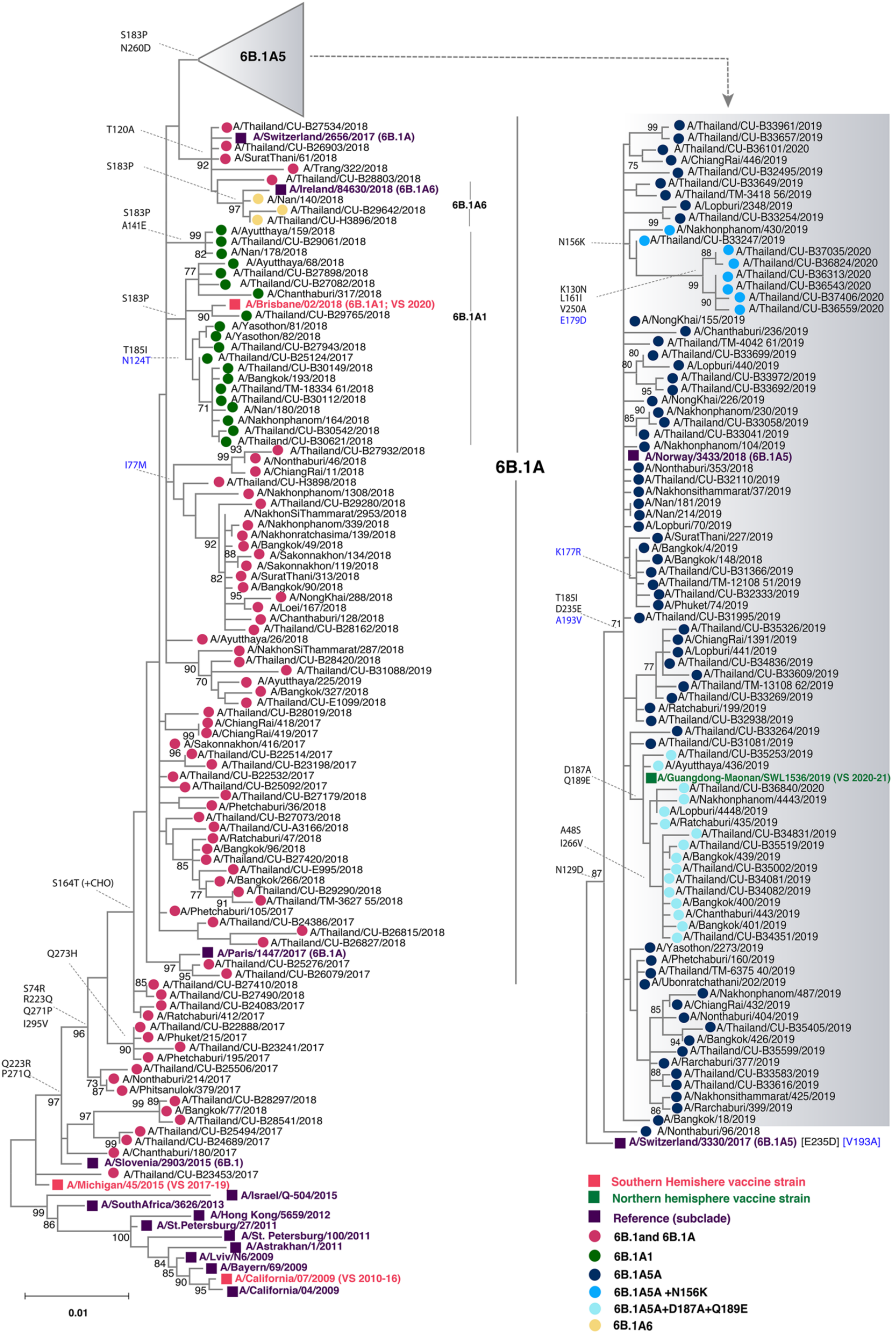
Phylogenetic analysis of the entire HA gene of A(H1N1)pdm09. Sequences from 90 A/Thailand/CU strains and 93 other Thai strains available from the database were compared with the A(H1N1)pdm09 vaccine and reference strains of known clades. Phylogenetic tree was constructed using the maximum-likelihood method using the TN93 + G model with 1,000 bootstrap replicates implemented in MEGAX. Branch values of > 70% are indicated at the nodes, along with the characteristic residues defining these branches. Residue changes in HA1 are denoted in black; changes in HA2 are in blue. Gain (+ CHO) and loss (−CHO) potential glycosylation sites were indicated. Southern hemisphere vaccine strains (magenta squares), the 2020–21 northern hemisphere vaccine (green squares) and the reference strains (purple squares) are indicated. Colored circles indicate the clade and subclade of A(H1N1)pdm09 circulating strains. VS defined as vaccine strain.

To better understand the genetic changes among the recently circulating influenza strains in Thailand, we selected representative influenza samples for genetic characterization (Table S1). We analyzed the phylogenetic tree of 183 HA sequences of influenza A(H1N1)pdm09 comprising the Thai strains identified in this study and previously elsewhere in Thailand, along with the vaccine and reference strains. Compared to A/Michigan/45/2015, the vaccine strain for the southern hemisphere from 2017 and 2019, all of the A(H1N1)pdm09 Thai strains belonged to clade 6B.1 and 89.6% (164/183) further clustered into subclade 6B.1A, which possessed changes of amino acid residue S74R, S164T, and I295V (Fig. 2). Since 2018, circulating Thai strains further diverged to form subclades 6B.1A1, 6B.1A5 and 6B.1A6 which all carried the S183P mutation (Fig. S1). Strains in the subclade 6B.1A5A (characterized by N129D, T185I, D235E, and D260N on HA1, and A193V on HA2) predominated in the 2019–2020 season. Interestingly, a subset of 6B.1A5A Thai strains possessed additional D187A and Q189E changes and clustered with the 2020–2021 season northern hemisphere vaccine strain A/Guangdong-Maonan/SWL1536/2019. The most divergent among the 6B.1A5A subclade was a cluster of strains possessing N156K, K130N, L161I, V250A (HA1) and E179D (HA2). Taken together, these data suggest that the existing A(H1N1)pdm09 strains circulating in recent years have genetically drifted from the A/Brisbane/02/2018, which was introduced as the 2020 southern hemisphere vaccine strain (Fig. S2).

#### A(H3N2)

**Figure 3:**
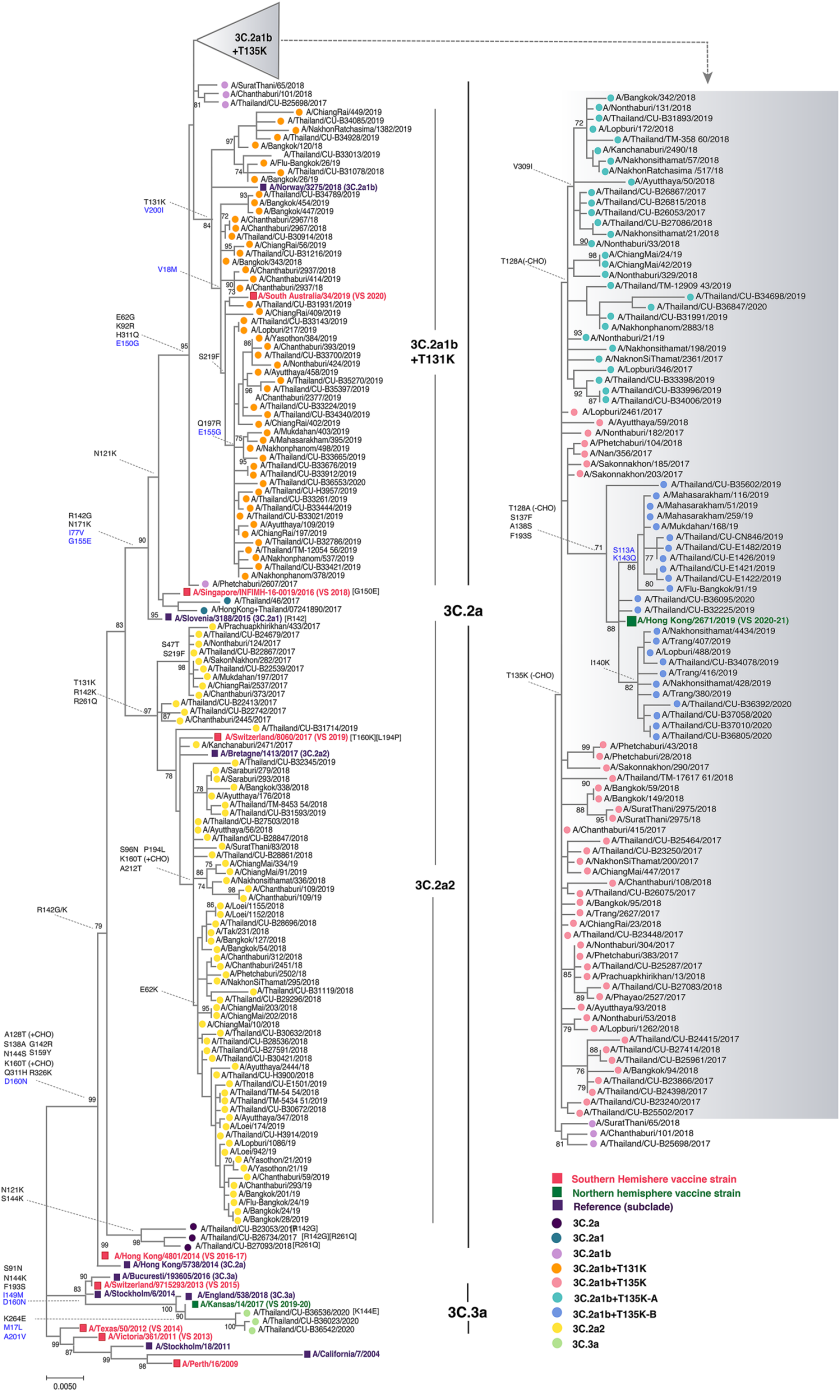
Phylogenetic analysis of the HA gene of A(H3N2). Sequences from 90 A/Thailand/CU strains and 142 other Thai strains available from the database were compared with the A(H3N2) vaccine and reference strains of known clades. Phylogenetic tree was constructed using the maximum-likelihood method using the HKY + G + I model with 1000 bootstrap replicates implemented in MEGAX. Branch values of > 70% are indicated at the nodes, along with the characteristic residues defining these branches. Residue changes in HA1 are denoted in black; changes in HA2 are in blue. Gain (+ CHO) and loss (−CHO) potential glycosylation sites were indicated. Southern hemisphere vaccine strains (magenta squares), the 2019–2021 northern hemisphere vaccine (green squares) and the reference strains (purple squares) are indicated. Colored circles denote the clade and subclade of A(H3N2) circulating strains. VS defined as vaccine strain.

Phylogenetic analysis of 232 HA sequences of influenza A(H3N2) comprising the Thai strains identified in this study and previously elsewhere in Thailand, along with the vaccine and reference strains, showed that 98.7% (229/232) belonged to clade 3C.2a (the remaining three Thai strains were 3C.3a, all identified in 2020) (Fig. 3). These Thai strains diverged into subclades 3C.2a1 (defined by N121K, R142G, and N171K on HA1, and I77V and G155E on HA2) and 3C.2a2 (defined by T131K, R142K, and R261Q on HA1) (Fig. S1). Further divergence into 3C.2a1b was marked by E62G, K92R, and H311Q on HA1, and E150G on HA2. Strains identified circulating during 2017–2018 are the 3C.2a1b with addition of T135K that circulated together with 3C.2a2 virus. Most circulating strains in 2019 were 3C.2a1b with T131K, which diverged from the 2019 season southern hemisphere vaccine strain A/Switzerland/8060/2017 (3C.2a2). Co-circulation of 3C.2a1b (with additional T135K and T128A) and 3C.3a virus was identified in the first three months of 2020. The additional residue changes in subclade 3C.2a1b at positions 128 and 135 resulted in a loss of glycosylation at these sites. The 3C.3a virus was characterized by S91N, N144K, F193S on HA1, and I149M and D160N on HA2 (by comparison with A/Switzerland/9,715,293/2013), which were genetically similar to the 2019–2020 season northern hemisphere vaccine strain A/Kansas/14/2017. From our data, A/South Australia/34/2019 chosen for the 2020 A(H3N2) southern hemisphere vaccine strain will not expect a good match since 60% of the currently circulating strains are from 3C.2a1 with T135K and T128A, while 30% are 3C.3a in 2020 (Fig. S2).

#### Influenza B virus

**Figure 4:**
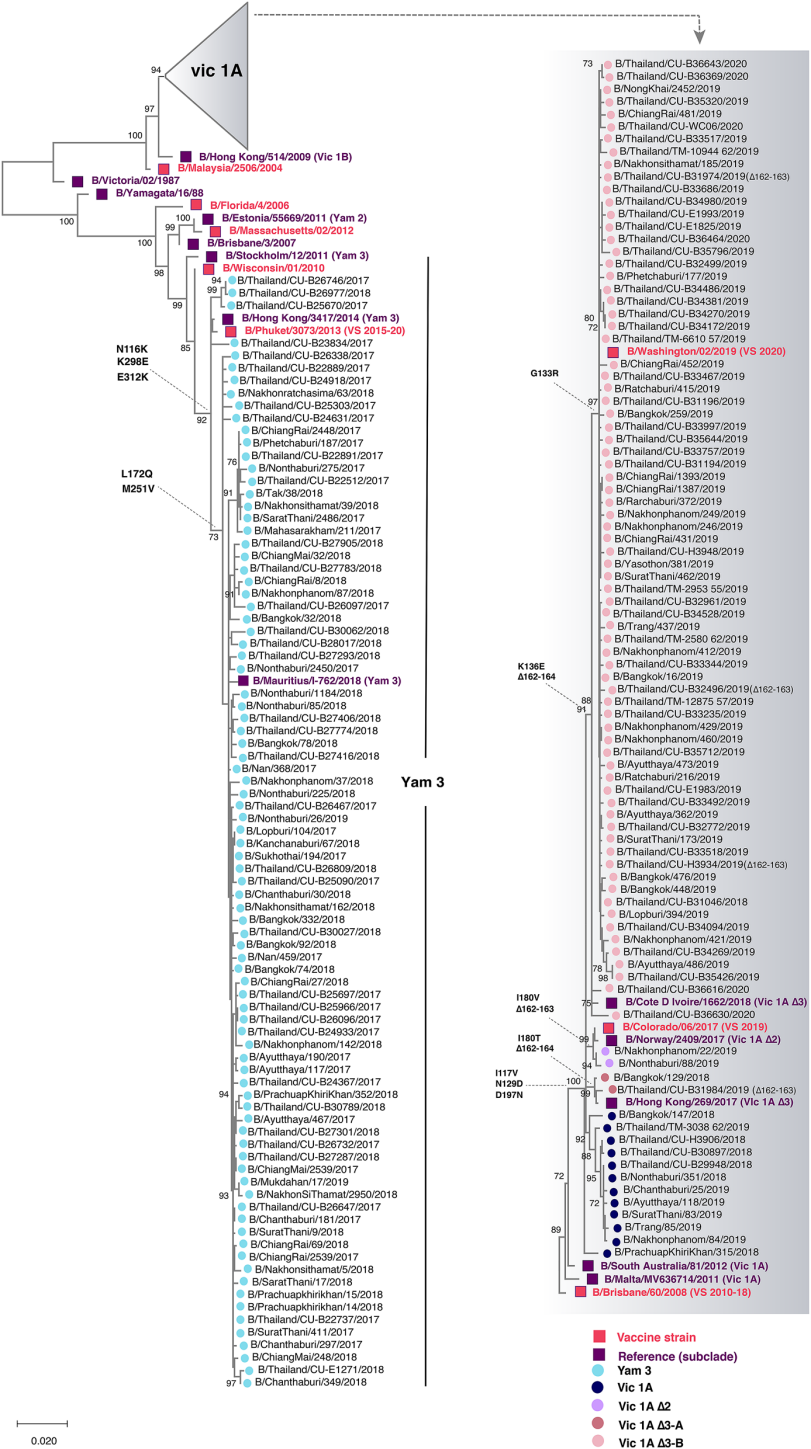
Phylogenetic analyses of the nucleotide sequences of the HA coding region of the influenza B virus. Eighty-one nucleotide sequences from this study (designated A/Thailand/CU) and other Thai strains available from the database during July 2017-March 2020 (n = 96) were compared with the influenza B vaccine and reference strains of known clades (magenta and purple squared, respectively). Phylogenetic tree was constructed using the maximum-likelihood method and the HKY + G model with 1000 bootstrap replicates implemented in MEGAX. Branch values of > 70% are indicated at nodes. Colored circles denote the clade and subclade of the circulating influenza B virus strains. VS defined as vaccine strain.

From the analysis of 177 HA sequences, the circulating B/Yamagata strains were belonged to clade 3 and experience very little change (L172Q and M251V) compared to the B/Phuket/3073/2013, which served as the southern hemisphere vaccine strain since 2015 (Fig. 4). Our B/Victoria strains belonged to clade 1A and grouped with B/Brisbane/60/2008, which was the vaccine strain prior to 2019. These strains possessed either 162–163 double deletions with I180V, or 162–164 triple deletions with I180T or K136E in HA1. From 2019 onward, however, strains with triple deletions and K136E predominated (68/77), which clustered with the 2020 southern hemisphere vaccine strain B/Washington/02/2019.

### Deduced amino acid changes mapped onto the receptor binding and antigenic sites of HA

**Figure 5:**
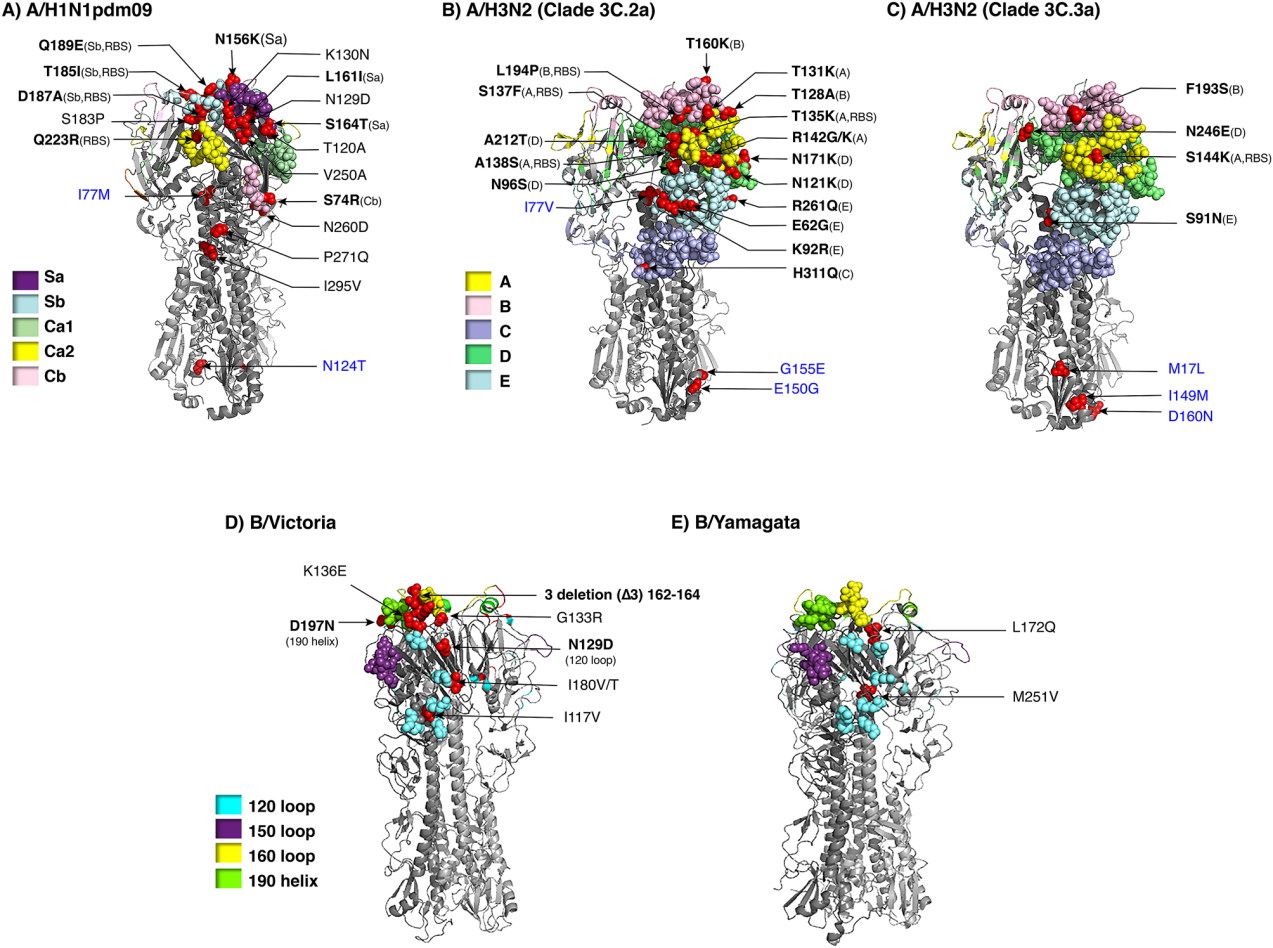
Placement of residue changes identified in the influenza virus strains in this study. Trimeric HA of influenza A and B virus for which three-dimensional structures were available from the Protein Data Bank (https://www.rcsb.org) served to illustrate differences in amino acids at various positions between the vaccine and the Thai strains identified in this study. The AA changes were mapped by using PyMOL Molecular Graphics System Version 1.3 (Schrödinger; https://www.schrodinger.com). A) The AA changes between A/Michigan/45/2015 and A(H1N1)pdm09 Thai strains were mapped onto HA structure of A/California/04/2009 (PDB:3LZG). Amino acid substitutions of A(H3N2) Thai strains compared with B) A/Hong Kong/4801/2014 (3C.2a) and C) A/Switzerland/9,715,293/2013 (3C.3a) were mapped on A/Victoria/361/2011 (PDB: 4WE9). D) Residue changes between B/Victoria Thai strains and vaccine strain, B/Brisbane/60/2008 (PDB: 4FQM) were shown. E) The AA changes of B/Yamagata Thai strains and B/Phuket/3073/2013 were mapped on B/Yamanashi/166/98 (PDB: 4M44). RBS and antigenic sites are color-coded. Residue of the vaccine strain, followed by the numerical position and the residue found in the Thai strains, are indicated with arrows pointing to the location (red). Bolded amino acid designations denote important antigenic and/or RBS, while blue designations are on HA2. Only one subunit comprising the trimeric HA is labeled for clarity.

To visualize how amino acid changes present among the Thai strains in this study may affect important domains on the HA, we used publicly available influenza A and B virus HA structures to project amino acid differences between the vaccine and the Thai strains. Comparing the circulating influenza A(H1N1)pdm09 with A/Michigan/45/2015 (vaccine strain since 2017), we identified N156K, L161I and S164T substitutions on the Sa antigenic site (Fig. 5A), which often drives A(H1N1)pdm09 evolution, particularly at residue 156^13^. We found that T185I, D187A, and Q189E mapped to the Sb antigenic site and overlapped the RBS, while S74R is on epitope Cb. No residue changes appeared on epitopes Ca1 nor Ca2.

Using A/Hong Kong/4801/2014 as a representative A(H3N2) vaccine strain for clade 3C.2a, five residue changes mapped to antigenic epitope A (T131K, T135K, S137F, A138S, R142G/K), three residues mapped to epitope B (T128A, T160K, and L194P), one residue mapped to epitope C (H311Q), four residues mapped to epitope D (N96S, N121K, N171K, and A212T), and three residues mapped to epitope E (E62G, K92R, and R261Q) (Fig. 5B). Using A/Switzerland/9,715,293/2013 as a representative of A(H3N2) vaccine strain for subclade 3C.3a, which was the vaccine strain in 2015, four residue changes identified among the Thai strains were mapped onto the HA structure (S144K on site A, F193S on site B, N246E on site D, and S91N on site E) (Fig. 5C). Five residues at positions 135, 137, 138, 144 and 194 varied on the RBS. Overall, most circulating A(H3N2) strains showed dominant diversity on epitope sites A, B, D and E.

On the HA structure of B/Brisbane/60/2008, circulating B/Victoria strains possessed substitutions at residues I117V, N129D, and K136E, which aggregate in the vicinity of the 120 loop region (Fig. 5D). Meanwhile, D197N which is a reversion of the substitution in the vaccine strain acquired as a result of egg-adaption was mapped to the 190 helix region. In contrast, none of the residue changes identified among the circulating B/Yamagata strains occurred on the antigenic or the RBS (Fig. 5E).

### Determination of the selection pressure on influenza virus

Amino acid changes on the HA often result from selective pressure being exerted on the virus by the host immunity during infection. We therefore evaluated the potential positive selection of these residues by examining their rate of change (d*N*/d*S*) (Table S2). The relative rate of d*N*/d*S* for overall sites under positive selection on the HA1 region was 0.194 for A(H1N1)pdm09, 0.228 for A(H3N2), 0.657 for B/Victoria lineage, and 0.0525 for B/Yamagata lineage. These rates of < 1 implied purifying selection which eliminates or hinders the spread of deleterious mutations. Site-by-site selection analysis showed that two residues at HA1 positions 120 and 233 in A(H1N1)pdm09 circulating strains were under positive selection. Meanwhile, HA1 residues 57,131, 135, 144, and 193 were positively selected in A(H3N2). Although mixed effects model of evolution (MEME) algorithm yielded 10 positive sites in B/Victoria (76, 80, 87, 121, 126, 128, 136, 154, 160, and 238), only residue 238 was also identified by the fixed-effects likelihood (FEL) method. For B/Yamagata strains, only residue 227 was positively selected. This result indicate that more diversity and less purifying selection controls HA1.

### Implications on the computed antigenic relatedness

**Figure 6:**
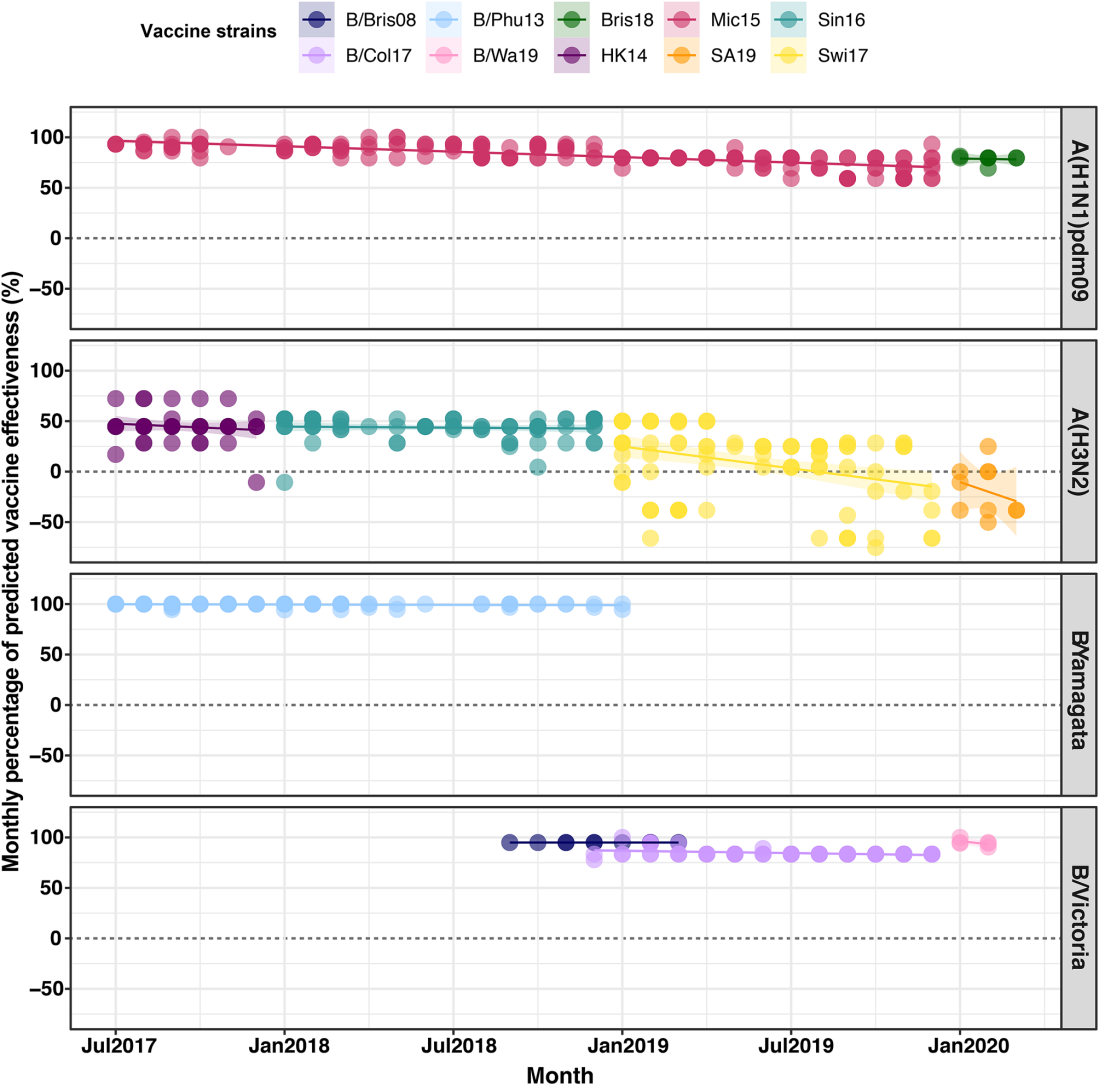
Estimation of the trend of computed antigenic relatedness between July 2017 and March 2020. Computed antigenic relatedness was derived using the *P*_epitope_ model by comparing vaccine strains and plotted by using R v3.6.0 (https://www.r-project.org). Circulating A(H1N1)pdm09 viruses were compared with A/Michigan/45/2015 (Mic15) and B/Brisbane/02/2018 (Bris18). Circulating A(H3N2) viruses were compared with A/Hong Kong/4801/2014 (HK14), A/Singapore/INFIMH-16–0019/2016 (Sin16), A/Switzerland/8060/2017 (Swi17) and A/South Australia/34/2019 (SA19). The circulating B/Yamagata-like lineage was compared with B/Phuket/3073/2013 (B/Phu13) and the B/Victoria-like lineage was compared with B/Brisbane/60/2008 (B/Bris08), B/Colorado/06/2017 (B/Col17), B/Washington/02/2019 (B/Wa19) with the corresponding number of deletions. The colored circles of each panel represent individual computed antigenic relatedness against the vaccine strain in that year.

We next evaluated how the evolving changes on the HA residues potentially affected antigenic relatedness (Table S3-S5). Given the genetic sequence of the A(H1N1)pdm09 strains identified in this study compared to the vaccine strain A/Michigan/45/2015 (which remained unchanged from 2017–2019), the computed antigenic relatedness gradually decreased from 91.7% in 2017, 88.3% in 2018, and 74.2% in 2019 (Fig. 6). These findings suggest that the recent A(H1N1)pdm09 Thai strains identified in this study have significantly drifted from the vaccine sequence (p < 0.001). For the new 2020 season A(H1N1)pdm09 southern hemisphere vaccine strain A/Brisbane/02/2018, however, the computed antigenic relatedness was 78.5% (Fig. S3). Except for 2017, the vaccine component for southern hemisphere A(H3N2) has changed yearly. From 2017–2020, the computed antigenic relatedness for A(H3N2) were 44.7%, 43.6%, 9.2%, and − 18.9%. These values suggest that the A(H3N2) vaccine strain was a relatively poor match for the circulating strains in Thailand during the past four years (p < 0.01).

On the other hand, the computed antigenic relatedness against the B/Victoria lineage strains for 2018 and 2019 were 94.9% and 85.8%, respectively (p < 0.001). For 2020, the chosen vaccine strain B/Washington/02/2019 demonstrated an improved antigenic relatedness of 94.8%. Finally, the fact that computed antigenic relatedness remains relatively high for the B/Yamagata vaccine strain B/Phuket/3073/2013 (94.2–100%), which has remained unchanged since 2015, suggests that the current vaccine strain remains appropriate. Taken together, computed antigenic relatedness estimates provided insight into the importance of the vaccine component match with the circulating influenza virus strains in the region.

## Discussion

Year-round influenza activity in Thailand can give rise to antigenically drifted influenza virus strains, which are sufficiently different to be categorized into emerging new subclades. These strains sometimes differ significantly from the vaccine strains and are able to escape the host immunity elicited by the annual influenza vaccine. Consequently, the effectiveness of influenza immunization is substantially reduced^3,35^. Our study aimed to characterize the predominantly circulating A(H1N1)pdm09 and A(H3N2), as well as both lineages of the influenza B virus and how residue changes can affect the antigenic relatedness. Analysis of their HA gene sequence and deduced amino acids identified genetic heterogeneity, which were different from those found in the southern hemisphere vaccine strains for their respective years.

Among the influenza A(H1N1)pdm09 virus strains in this study, most amino acid substitutions contributing to the phylogenetic cluster transitions were accumulated at the antigenic sites Sa and Sb, while A(H3N2) demonstrated greater diversity with most mutations dominantly located on epitopes A, B, D and E. Meanwhile, antigenic drifts of the influenza B virus occurred at the 120 loop and the 190 helix. Observed changes on the antigenic and the RBS can be important since even one residue substitution at these sites could potentially drive new antigenic variants^12,36^.

The computed antigenic relatedness based on the HA of A(H1N1)pdm09 decreased significantly in 2019 from the previous year. This suggests that most circulating influenza strains were antigenically different from the A/Michigan/45/2015 vaccine component for 2019 and the A/Brisbane/02/2018 vaccine component for 2020. The A(H1N1)pdm09 strains predominant in Thailand from 2010 to 2015 season belonged primarily to clade 6B.1^27^, for which A/Michigan/45/2015 is a member and has served as the vaccine strain from 2017 to 2019. Since then, more recent A(H1N1)pdm09 strains have acquired S74R, S164T, and I295V, which have evolved into subclade 6B.1A^17^. Some strains with an additional S183P adjacent to the Sb antigenic site now form subclades 6B.1A1 to 6B.1A7^8^. Several of the Thai A(H1N1)pdm09 circulating in 2018 are 6B.1A1 and 6B.1A6 strains, which is noteworthy since experimental studies have shown that serum from those immunized with the vaccine strain of clade 6B.1 was unable to efficiently neutralize influenza virus strain carrying S183P. This was why B/Brisbane/02/2018 (subclade 6B.1A1) was chosen for the 2020 flu season^22^. From 2019 onward, the majority of the A(H1N1)pdm09 Thai strains was 6B.1A5A, which was the predominant subclade in the 2018–2019 influenza season in Europe^37^. Several studies of vaccine estimates among all ages in 2018–2019 influenza season revealed that the predominant newly subclade was associated with a relatively low VE (A/Michigan/45/2015) against influenza A(H1N1)pdm09, which found in Europe (40–71%), USA( 30–58%), Canada (72%)^38–40^. Although B/Brisbane/02/2018 was used in the 2019–2020 influenza season for the northern hemisphere, low VE against A(H1N1)pdm09 has been observed in the USA (37%) and Europe (48–75%)^41,42^. Thus, 6B.1A5A strains in this study, however, possessed the additional D189E and either N156K or D187A. These changes, if continued, are expected to result in the introduction of a new strain component for the 2021 influenza vaccine.

The computed antigenic relatedness for A(H3N2) during this study period, on the other hand, performed worse than A(H1N1)pdm09. This was not surprising given that the evolutionary rate for A(H3N2) in the HA1 domain is considerably greater than that for A(H1N1)pdm09^43^. Among several circulating A(H3N2), clade 3C.2a predominated since 2015^27^ and has now accumulated sufficient changes to be classified as 3C.2a1 and 3C.2a2^17^. Since then, 3C.2a1b was established, which has gained additional substitutions (either T131K or T135K with T128A) in HA1 and these changes are located in the antigenic and the RBS region^7^. The 3C.2a1b virus was also reportedly the predominant subclade in Europe for 2017–2018 winter month^37^. Due to multiple clades/subclades circulation, our study found the mismatch of the 2019 southern hemisphere vaccine (A/Switzerland/8060/2017 (subclade 3C.2a2)) and circulating strains in 2019 (3C.2a1b + T131K ). Furthermore, although A/South Australia/34/2019 which is a 3C.2a1b + T131K strain was announced for the 2020 southern hemisphere vaccine strain^22^, the predominate strains in the first three months of 2020 were subclade 3C.2a1b with additional T135K and T128A which slightly far away from their vaccine strain. T135K and T128A, which are located at antigenic sites A and B respectively, are predicted to cause a loss of glycosylation that might alter the HA antigenic properties and affect antibody recognition^6^. Further selective pressure analysis on the HA1 of A(H3N2) suggests that the residues changes at positions 131, 135, 144 and 193 have d*N*/d*S* ≥ 1 which indicated the positive selection due to immune escape mechanisms. Detection of clade 3C.3a strains in 2020 has been less frequent so far. A previous report showed that antibodies generated against 3C.2a less neutralize 3C.3a virus, particularly when 3C.3a possesses an additional F193S. It also does not neutralize 3C.2a1 well compared to 3C.2a2^44^.

The A(H3N2) vaccine strain was not effective during the 2018–19 influenza season in Europe, and low VE (− 58 to 57%) continues during 2019–20 season^39,42^. Our study also found that the computed antigenic relatedness against A(H3N2) was < 50% between 2019 and 2020 due to the circulation of multiple clades/subclades. These data suggested that the genetic diversity in A(H3N2) might hamper identification of a well-matched virus as a vaccine component in the 2014–2015 influenza season^45^ A meta-analysis indicated reduced protection and substantial variation of VE against A(H3N2). At the same time, the influenza vaccine provided moderate-to-high protection against A(H1N1)pdm09 and influenza B viruses^35^. Alternatives to egg-based manufacturing should proceed since a negative impact of egg-induced mutations in the H3N2 vaccine strain has been found^46^.

Newly emerging influenza B/Victoria strains are antigenically distinct and possess either double deletion at residues 162–163 or triple deletion at residues 162–164 within the HA1 domain. In this study, the triple deletion strains were more commonly found than the ancestor B/Brisbane-like strain during the 2019–2020 season. New B/Victoria lineages are actively circulating and warrant changing vaccine strain recommendations for this lineage since 2018^8,17^. Interim VE estimates of the 2019–2020 influenza season in the U.S. against the predominant B/Victoria lineage was 39–59% which showed a higher VE than influenza A virus^41^. This is consistent with a European study showing the VE of 62–83% against the influenza B virus for all ages during 2019–2020 season^42^. Our study also revealed a high computed antigenic relatedness against the new B/Victoria virus.

Control measures to mitigate coronavirus disease 2019 (COVID-19) pandemic have resulted in a decrease of influenza activity in 2020 thus far compared to the corresponding period of the previous year^47–49^. Monitoring influenza virus genetic drift remains critical in tracking novel residue changes, which could potentially affect virulence and evasion of host immunity.

Our study had several limitations. First, there was no information on the vaccination status of the individuals with the samples sent to us for influenza virus testing. Second, the results for computed antigenic relatedness, which was assessed using the accumulated substitutions on antigenic sites, ideally would require additional confirmatory antigenic characterization such as hemagglutinin inhibition or virus neutralization assay in order to complement and strengthen our existing data. Finally, we did not investigate the genetic changes in the neuraminidase gene, which encodes a surface glycoprotein that is also immunogenic.

By monitoring the genetic and antigenic changes of the influenza virus circulating in the tropics, data from this study suggests that new clades and subclades for both influenza A(H1N1)pmd09 and A(H3N2) viruses increasingly differ from those of the chosen vaccine strain, but not influenza B virus. These findings highlight the need for improved vaccine strain match particularly for A(H3N2).

## Supplementary Information

Supplementary Information.

## Data Availability

All data generated during this study are contained within this manuscript and its Supplementary Information files.
