# Supplementary material for: Characterizing genetic and antigenic divergence from vaccine strain of influenza A and B viruses circulating in Thailand, 2017–2020
Source: Sci Rep. 2021 Jan 12;11:735. doi: 10.1038/s41598-020-80895-w (PMC7803983; doi:10.1038/s41598-020-80895-w)
Supplement: Supplementary file 1 — Supplementary Information. [file 41598_2020_80895_MOESM1_ESM.pdf]

Characterizing genetic and antigenic divergence from vaccine strain of influenza A and B viruses circulating in Thailand, 2017-2020

Nungruthai Suntronwong, Sirapa Klinfueng, Sumeth Korkong, Preeyaporn Vichaiwattana, Thanunrat Thongmee, Sompong Vongpunsawad, Yong Poovorawan*

Supplementary Table 1. Accession numbers of HA sequences of circulating influenza A(H1N1)pdm09, A(H3N2) and B virus strains in this study.

Influenza A(H1N1)pdm09

Accession No.	Sequence ID	Accession No.	Sequence ID
MT803149	A/Thailand/CU-B28541/2018	MT803194	A/Thailand/CU-B33041/2019
MT803150	A/Thailand/CU-B28297/2018	MT803195	A/Thailand/CU-B33058/2019
MT803151	A/Thailand/CU-B25494/2017	MT803196	A/Thailand/CU-B32333/2019
MT803152	A/Thailand/CU-B24689/2017	MT803197	A/Thailand/CU-B33247/2019
MT803153	A/Thailand/CU-B23453/2017	MT803198	A/Thailand/CU-B32110/2019
MT803154	A/Thailand/CU-B23241/2017	MT803199	A/Thailand/CU-B31995/2019
MT803155	A/Thailand/CU-B22888/2017	MT803200	A/Thailand/CU-B31366/2019
MT803156	A/Thailand/CU-B25506/2017	MT803201	A/Thailand/CU-B27932/2018
MT803157	A/Thailand/CU-B31088/2019	MT803202	A/Thailand/CU-B28803/2018
MT803158	A/Thailand/CU-B28420/2018	MT803203	A/Thailand/CU-B26903/2018
MT803159	A/Thailand/CU-E1099/2018	MT803204	A/Thailand/CU-B27534/2018
MT803160	A/Thailand/CU-B36824/2020	MT803205	A/Thailand/CU-B29642/2018
MT803161	A/Thailand/CU-B37035/2020	MT803206	A/Thailand/CU-H3896/2018
MT803162	A/Thailand/CU-B36559/2020	MT803207	A/Thailand/CU-B29280/2018
MT803163	A/Thailand/CU-B36313/2020	MT803208	A/Thailand/CU-B28162/2018
MT803164	A/Thailand/CU-B36543/2020	MT803209	A/Thailand/CU-B25276/2017
MT803165	A/Thailand/CU-B37406/2020	MT803210	A/Thailand/CU-B26079/2017
MT803166	A/Thailand/CU-B33254/2019	MT803211	A/Thailand/CU-B26815/2018
MT803167	A/Thailand/CU-B36101/2020	MT803212	A/Thailand/CU-B26827/2018
MT803168	A/Thailand/CU-B33657/2019	MT803213	A/Thailand/CU-B27179/2018
MT803169	A/Thailand/CU-B33961/2019	MT803214	A/Thailand/CU-B29765/2018
MT803170	A/Thailand/CU-B32495/2019	MT803215	A/Thailand/CU-B29061/2018

Accession No.	Sequence ID	Accession No.	Sequence ID
MT803171	A/Thailand/CU-B33264/2019	MT803216	A/Thailand/CU-E995/2018
MT803172	A/Thailand/CU-B35405/2019	MT803217	A/Thailand/CU-B29290/2018
MT803173	A/Thailand/CU-B34831/2019	MT803218	A/Thailand/CU-B27420/2018
MT803174	A/Thailand/CU-B34351/2019	MT803219	A/Thailand/CU-A3166/2018
MT803175	A/Thailand/CU-B35002/2019	MT803220	A/Thailand/CU-B25124/2017
MT803176	A/Thailand/CU-B34081/2019	MT803221	A/Thailand/CU-B30149/2018
MT803177	A/Thailand/CU-B34082/2019	MT803222	A/Thailand/CU-B30542/2018
MT803178	A/Thailand/CU-B35519/2019	MT803223	A/Thailand/CU-B30621/2018
MT803179	A/Thailand/CU-B36840/2020	MT803224	A/Thailand/CU-B30112/2018
MT803180	A/Thailand/CU-B35253/2019	MT803225	A/Thailand/CU-B27943/2018
MT803181	A/Thailand/CU-B33609/2019	MT803226	A/Thailand/CU-B27082/2018
MT803182	A/Thailand/CU-B33269/2019	MT803227	A/Thailand/CU-B27898/2018
MT803183	A/Thailand/CU-B35326/2019	MT803228	A/Thailand/CU-B24386/2017
MT803184	A/Thailand/CU-B34836/2019	MT803229	A/Thailand/CU-H3898/2018
MT803185	A/Thailand/CU-B35599/2019	MT803230	A/Thailand/CU-B28019/2018
MT803186	A/Thailand/CU-B33692/2019	MT803231	A/Thailand/CU-B27073/2018
MT803187	A/Thailand/CU-B33972/2019	MT803232	A/Thailand/CU-B22514/2017
MT803188	A/Thailand/CU-B33649/2019	MT803233	A/Thailand/CU-B23198/2017
MT803189	A/Thailand/CU-B33583/2019	MT803234	A/Thailand/CU-B25092/2017
MT803190	A/Thailand/CU-B33616/2019	MT803235	A/Thailand/CU-B22532/2017
MT803191	A/Thailand/CU-B32938/2019	MT803236	A/Thailand/CU-B27410/2018
MT803192	A/Thailand/CU-B33699/2019	MT803237	A/Thailand/CU-B27490/2018
MT803193	A/Thailand/CU-B31081/2019	MT803238	A/Thailand/CU-B24083/2017

Influenza A(H3N2) virus

Accession No.	Sequence ID	Accession No.	Sequence ID
MT803239	A/Thailand/CU-B36536/2020	MT803284	A/Thailand/CU-B24398/2017
MT803240	A/Thailand/CU-B36023/2020	MT803285	A/Thailand/CU-B27414/2018
MT803241	A/Thailand/CU-B36542/2020	MT803286	A/Thailand/CU-B25961/2017
MT803242	A/Thailand/CU-B35602/2019	MT803287	A/Thailand/CU-B31991/2019
MT803243	A/Thailand/CU-CN846/2019	MT803288	A/Thailand/CU-B25464/2017
MT803244	A/Thailand/CU-E1482/2019	MT803289	A/Thailand/CU-B33398/2019
MT803245	A/Thailand/CU-E1426/2019	MT803290	A/Thailand/CU-B33996/2019
MT803246	A/Thailand/CU-E1421/2019	MT803291	A/Thailand/CU-B34006/2019
MT803247	A/Thailand/CU-E1422/2019	MT803292	A/Thailand/CU-B31893/2019
MT803248	A/Thailand/CU-B36095/2020	MT803293	A/Thailand/CU-B26867/2017
MT803249	A/Thailand/CU-B32225/2019	MT803294	A/Thailand/CU-B26053/2017
MT803250	A/Thailand/CU-B36392/2020	MT803295	A/Thailand/CU-B26815/2018
MT803251	A/Thailand/CU-B37010/2020	MT803296	A/Thailand/CU-B27086/2018
MT803252	A/Thailand/CU-B36805/2020	MT803297	A/Thailand/CU-B27083/2018

Accession No.	Sequence ID	Accession No.	Sequence ID
MT803253	A/Thailand/CU-B37058/2020	MT803298	A/Thailand/CU-B23250/2017
MT803254	A/Thailand/CU-B34078/2019	MT803299	A/Thailand/CU-B23240/2017
MT803255	A/Thailand/CU-B33013/2019	MT803300	A/Thailand/CU-B25287/2017
MT803256	A/Thailand/CU-B31078/2018	MT803301	A/Thailand/CU-B26075/2017
MT803257	A/Thailand/CU-B34085/2019	MT803302	A/Thailand/CU-B25502/2017
MT803258	A/Thailand/CU-B34928/2019	MT803303	A/Thailand/CU-B23448/2017
MT803259	A/Thailand/CU-B34789/2019	MT803304	A/Thailand/CU-B23053/2017
MT803260	A/Thailand/CU-B36553/2020	MT803305	A/Thailand/CU-B26734/2017
MT803261	A/Thailand/CU-B31931/2019	MT803306	A/Thailand/CU-B27093/2018
MT803262	A/Thailand/CU-B30914/2018	MT803307	A/Thailand/CU-B31119/2018
MT803263	A/Thailand/CU-B31216/2019	MT803308	A/Thailand/CU-B31714/2019
MT803264	A/Thailand/CU-B35270/2019	MT803309	A/Thailand/CU-B32345/2019
MT803265	A/Thailand/CU-B35397/2019	MT803310	A/Thailand/CU-B31593/2019
MT803266	A/Thailand/CU-B34340/2019	MT803311	A/Thailand/CU-B29296/2018
MT803267	A/Thailand/CU-B32786/2019	MT803312	A/Thailand/CU-B30632/2018
MT803268	A/Thailand/CU-B33665/2019	MT803313	A/Thailand/CU-B28861/2018
MT803269	A/Thailand/CU-H3957/2019	MT803314	A/Thailand/CU-E1501/2019
MT803270	A/Thailand/CU-B33676/2019	MT803315	A/Thailand/CU-H3900/2018
MT803271	A/Thailand/CU-B33912/2019	MT803316	A/Thailand/CU-B30672/2018
MT803272	A/Thailand/CU-B33421/2019	MT803317	A/Thailand/CU-H3914/2019
MT803273	A/Thailand/CU-B33444/2019	MT803318	A/Thailand/CU-B28696/2018
MT803274	A/Thailand/CU-B33261/2019	MT803319	A/Thailand/CU-B28847/2018
MT803275	A/Thailand/CU-B33143/2019	MT803320	A/Thailand/CU-B27591/2018
MT803276	A/Thailand/CU-B33021/2019	MT803321	A/Thailand/CU-B30421/2018
MT803277	A/Thailand/CU-B33700/2019	MT803322	A/Thailand/CU-B27503/2018
MT803278	A/Thailand/CU-B33224/2019	MT803323	A/Thailand/CU-B28536/2018
MT803279	A/Thailand/CU-B34698/2019	MT803324	A/Thailand/CU-B24679/2017
MT803280	A/Thailand/CU-B25698/2017	MT803325	A/Thailand/CU-B22867/2017
MT803281	A/Thailand/CU-B36847/2020	MT803326	A/Thailand/CU-B22539/2017
MT803282	A/Thailand/CU-B24415/2017	MT803327	A/Thailand/CU-B22742/2017
MT803283	A/Thailand/CU-B23866/2017	MT803328	A/Thailand/CU-B22413/2017

Influenza B virus

Accession No.	Sequence ID	Accession No.	Sequence ID
MT803397	B/Thailand/CU-H3906/2018	MT803438	B/Thailand/CU-B31196/2019
MT803398	B/Thailand/CU-B30897/2018	MT803439	B/Thailand/CU-B33467/2019
MT803399	B/Thailand/CU-B29948/2018	MT803440	B/Thailand/CU-B34528/2019
**Accession No.**	**Sequence ID**	**Accession No.**	**Sequence ID**
MT803400	B/Thailand/CU-B31984/2019	MT803441	B/Thailand/CU-B35712/2019

MT803401	B/Thailand/CU-B36630/2020	MT803442	B/Thailand/CU-B23834/2017
MT803402	B/Thailand/CU-B36616/2020	MT803443	B/Thailand/CU-B22512/2017
MT803403	B/Thailand/CU-B35426/2019	MT803444	B/Thailand/CU-B22891/2017
MT803404	B/Thailand/CU-B35796/2019	MT803445	B/Thailand/CU-B25303/2017
MT803405	B/Thailand/CU-B34172/2019	MT803446	B/Thailand/CU-B26338/2017
MT803406	B/Thailand/CU-B34270/2019	MT803447	B/Thailand/CU-B24631/2017
MT803407	B/Thailand/CU-E1993/2019	MT803448	B/Thailand/CU-B22889/2017
MT803408	B/Thailand/CU-WC06/2020	MT803449	B/Thailand/CU-B24918/2017
MT803409	B/Thailand/CU-B35320/2019	MT803450	B/Thailand/CU-B26097/2017
MT803410	B/Thailand/CU-B34269/2019	MT803451	B/Thailand/CU-B27905/2018
MT803411	B/Thailand/CU-B32496/2019	MT803452	B/Thailand/CU-B27783/2018
MT803412	B/Thailand/CU-B33492/2019	MT803453	B/Thailand/CU-B27406/2018
MT803413	B/Thailand/CU-E1825/2019	MT803454	B/Thailand/CU-B27774/2018
MT803414	B/Thailand/CU-B33997/2019	MT803455	B/Thailand/CU-B27416/2018
MT803415	B/Thailand/CU-B34094/2019	MT803456	B/Thailand/CU-B30062/2018
MT803416	B/Thailand/CU-B31974/2019	MT803457	B/Thailand/CU-B27293/2018
MT803417	B/Thailand/CU-B33686/2019	MT803458	B/Thailand/CU-B30789/2018
MT803418	B/Thailand/CU-B33757/2019	MT803459	B/Thailand/CU-E1271/2018
MT803419	B/Thailand/CU-B36464/2020	MT803460	B/Thailand/CU-B28017/2018
MT803420	B/Thailand/CU-B34980/2019	MT803461	B/Thailand/CU-B30027/2018
MT803421	B/Thailand/CU-B36369/2020	MT803462	B/Thailand/CU-B26467/2017
MT803422	B/Thailand/CU-B36643/2020	MT803463	B/Thailand/CU-B24367/2017
MT803423	B/Thailand/CU-B34381/2019	MT803464	B/Thailand/CU-B27287/2018
MT803424	B/Thailand/CU-E1983/2019	MT803465	B/Thailand/CU-B27301/2018
MT803425	B/Thailand/CU-B33344/2019	MT803466	B/Thailand/CU-B26732/2017
MT803426	B/Thailand/CU-H3948/2019	MT803467	B/Thailand/CU-B25966/2017
MT803427	B/Thailand/CU-B33517/2019	MT803468	B/Thailand/CU-B26096/2017
MT803428	B/Thailand/CU-B32499/2019	MT803469	B/Thailand/CU-B25697/2017
MT803429	B/Thailand/CU-B35644/2019	MT803470	B/Thailand/CU-B24933/2017
MT803430	B/Thailand/CU-B32772/2019	MT803471	B/Thailand/CU-B25090/2017
MT803431	B/Thailand/CU-B34486/2019	MT803472	B/Thailand/CU-B26809/2018
MT803432	B/Thailand/CU-B33518/2019	MT803473	B/Thailand/CU-B26647/2017
MT803433	B/Thailand/CU-B33235/2019	MT803474	B/Thailand/CU-B22737/2017
MT803434	B/Thailand/CU-B32961/2019	MT803475	B/Thailand/CU-B25670/2017
MT803435	B/Thailand/CU-B31194/2019	MT803476	B/Thailand/CU-B26746/2017
MT803436	B/Thailand/CU-H3934/2019	MT803477	B/Thailand/CU-B26977/2018
MT803437	B/Thailand/CU-B31046/2018		

Supplementary Table 2. Positive selected sites on the HA coding sequences of influenza A and B viruses identified in Thailand between July 2017 and March 2020.

**Subtype**	**Mixed Effects Model of Evolution**					**Fixed effects likelihood**		
	**d*N*/d*S*[a]**	**HA regions**	**position**	**β+**	**P-value[b]**	**position**	**omega**	**P-value[b]**
A/H1N1(pdm09)	0.194	HA1	**120**	3.77	0.05	**120**	Infinity	0.033
			233	1.76	0.09	233	Infinity	0.069
		HA2	213	3963.97	<0.001			
A/H3N2	0.228	HA1	57	12.75	0.06	57	Infinity	0.049
			**131**	1574.54	<0.001	**135**	Infinity	0.034
			**135**	7.49	0.05	**193**	Infinity	0.058
			**144**	53.68	0.07			
			**193**	3.21	0.08			
		HA2	18	1.94	0.09	18	Infinity	0.071
			200	3.25	0.06	200	Infinity	0.043
			201	2.43	0.1	201	Infinity	0.075
B/Victoria	0.657	HA1	76	168.8	0.03	238	Infinity	0.058
			80	150.56	0.05			
			87	96.25	<0.001			
			121	255.22	<0.001			
			126	32.72	0.04			
			128	247.49	0.04			
			136	37.85	0.04			
			154	154.28	0.03			
			160	44.19	0.03			
			238	1.8	0.08			
B/Yamagata	0.0525	HA1	227	3659.33	0.01	N/D	N/D	N/D

N/D defined as not detected.

[a] d*N*/d*S* is the ratio of non-synonymous/synonymous substitutions.

[b] P-value from the MEME and FEL result for a positive selection level.

β+ is the non-synonymous substitution rate at a site for the positive/neutral evolution component.

Bold text denotes an amino-acid substitution associated with a clade/subclade transition.

Supplementary Table 3. Computed antigenic relatedness among the vaccine strain and number of mutations found on the dominant epitope of circulating influenza A(H1N1)pdm09 in Thailand.

Year	Vaccine strain	No. of strains	Dominant epitope	Number of mutations	Pepitope	Efficacy	E (53%)	E (100%)
2017	A/Michigan/45/2015	2	No	0	0.000	0.530	53.00	100.00
		2	A	1	0.042	0.480	48.04	90.64
		4	B	1	0.045	0.476	47.59	89.79
		1	B	2	0.091	0.422	42.18	79.59
		13	C	1	0.030	0.494	49.39	93.20
		3	C	2	0.061	0.458	45.79	86.39
		1	D	1	0.021	0.505	50.52	95.32
		1	E	1	0.029	0.495	49.50	93.40
		2	E	2	0.059	0.460	46.00	86.79
2018	A/Michigan/45/2015	3	No	0	0.000	0.530	53.00	100.00
		4	A	1	0.042	0.480	48.04	90.64
		3	A	2	0.083	0.431	43.08	81.29
		12	B	1	0.045	0.476	47.59	89.79
		15	B	2	0.091	0.422	42.18	79.59
		21	C	1	0.030	0.494	49.39	93.20
		4	C	2	0.061	0.458	45.79	86.39
		1	C	3	0.091	0.422	42.18	79.59
		1	D	2	0.042	0.480	48.04	90.64
		1	E	1	0.029	0.495	49.50	93.40
		3	E	2	0.059	0.460	46.00	86.79
2019	A/Michigan/45/2015	1	A	3	0.125	0.381	38.13	71.93
		1	B	1	0.045	0.476	47.59	89.79
		46	B	2	0.091	0.422	42.18	79.59
		14	B	3	0.136	0.368	36.77	69.38
		14	B	4	0.182	0.314	31.36	59.18
		1	C	1	0.030	0.494	49.39	93.20
		1	E	4	0.118	0.390	39.00	73.58
2020	A/Brisbane/02/2018	1	A	2	0.083	0.431	43.08	81.29
		5	B	2	0.091	0.422	42.18	79.59
		1	B	3	0.136	0.368	36.77	69.38
		1	C	3	0.091	0.422	42.18	79.59

The positions of amino acid residues in the epitope were based on the Deem MW et al., 2009.
E (53%) was calculated from $E = -1.19 \times P_{epitope} + 0.53$ in which efficacy is 53% when the $P_{epitope} = 0$.
E (100%) = E (53%) *100 /53.

Supplementary Table 4. Estimation of computed antigenic relatedness between the vaccine strain and number of mutations found on the dominant epitope of circulating influenza A(H3N2) in Thailand.

Year	Vaccine strain	No. of strains	Dominant epitope	Number of mutations	Pepitope	Efficacy	E (47%)	E (100%)
2017	A/Hong Kong/4801/2014	5	A	1	0.053	0.340	34.00	72.34
		31	A	2	0.105	0.210	21.00	44.68
		1	A	3	0.158	0.080	8.00	17.02
		1	A	4	0.211	-0.050	-5.00	-10.64
		3	E	2	0.091	0.245	24.55	52.22
		6	E	3	0.136	0.133	13.32	28.34
2018	A/Singapore/INFIMH-16-0[illegible]	33	A	2	0.105	0.210	21.00	44.68
		1	A	4	0.211	-0.050	-5.00	-10.64
		3	B	2	0.095	0.235	23.48	49.95
		1	B	3	0.143	0.117	11.71	24.92
		7	C	3	0.111	0.196	19.56	41.61
		24	E	2	0.091	0.245	24.55	52.22
		9	E	3	0.136	0.133	13.32	28.34
		1	E	4	0.182	0.021	2.09	4.45
2019	A/Switzerland/8060/2017	6	A	3	0.158	0.080	8.00	17.02
		2	A	4	0.211	-0.050	-5.00	-10.64
		11	A	5	0.263	-0.180	-18.00	-38.30
		8	A	6	0.316	-0.310	-31.00	-65.96
		20	B	2	0.095	0.235	23.48	49.95
		19	B	3	0.143	0.117	11.71	24.92
		3	B	4	0.190	0.000	-0.05	-0.10
		1	B	7	0.333	-0.353	-35.33	-75.18
		1	D	4	0.098	0.229	22.90	48.73
		13	E	3	0.136	0.133	13.32	28.34
		8	E	4	0.182	0.021	2.09	4.45
		3	E	5	0.227	-0.091	-9.14	-19.44
		1	E	6	0.273	-0.204	-20.36	-43.33
2020	A/South Australia/34/2019	1	A	4	0.211	-0.050	-5.00	-10.64
		4	A	5	0.263	-0.180	-18.00	-38.30
		1	B	3	0.143	0.117	11.71	24.92
		3	B	4	0.190	0.000	-0.05	-0.10
		1	B	6	0.286	-0.236	-23.57	-50.15

The position of amino acid residues in the epitope was based on the Gupta V et al., 2006.
E(47%) was calculated from $E = -2.47 \times P_{epitope} + 0.47$ in which efficacy is 47% when the $P_{epitope} = 0$.
E (100%) = E (47%) *100 /47.

Supplementary Table 5. Computed antigenic relatedness among the vaccine strain and number of mutations found on the dominant epitope of circulating influenza B virus in Thailand.

Year	Vaccine strain	No. of strains	Dominant epitope	Number of mutations	Pepitope	Efficacy	E (68.24%)	E (100%)
	**B/Yamagata-lineages**							
2017	B/Phuket/3073/2013	34	No	0	0.000	0.682	68.24	100.00
		1	A	1	0.045	0.643	64.31	94.24
		1	B	1	0.042	0.646	64.61	94.72
		1	C	1	0.043	0.645	64.48	94.50
		2	D	1	0.025	0.661	66.08	96.83
2018	B/Phuket/3073/2013	39	No	0	0.000	0.682	68.24	100.00
		1	A	1	0.045	0.643	64.31	94.24
		1	B	1	0.042	0.646	64.61	94.72
		1	C	1	0.043	0.645	64.48	94.50
		3	D	1	0.025	0.661	66.08	96.83
2019	B/Phuket/3073/2013	2	No	0	0.000	0.682	68.24	100.00
	**B/Victoria-lineages**							
2018	B/Brisbane/60/2008	6	B	1	0.040	0.648	64.78	94.94
	B/Washington/02/2019	1 (Δ3)	No	0	0.000	0.682	68.24	100.00
		1 (Δ3)	B	1	0.045	0.643	64.31	94.24
2019	B/Brisbane/60/2008	6	B	1	0.040	0.648	64.78	94.94
	B/Colorado/06/2017	1 (Δ2)	No	0	0.000	0.682	68.24	100.00
		3 (Δ2)	A	1	0.045	0.643	64.31	94.24
		1 (Δ2)	B	1	0.043	0.645	64.48	94.50
		1 (Δ2)	E	1	0.040	0.648	64.78	94.94
	B/Washington/02/2019	52 (Δ3)	No	0	0.000	0.682	68.24	100.00
		9 (Δ3)	A	1	0.045	0.643	64.31	94.24
		1 (Δ3)	B	1	0.045	0.643	64.31	94.24
		3 (Δ3)	D	1	0.025	0.661	66.08	96.83
2020	B/Washington/02/2019	1 (Δ3)	No	0	0.000	0.682	68.24	100.00
		1 (Δ3)	A	1	0.045	0.643	64.31	94.24
		1 (Δ3)	B	1	0.045	0.643	64.31	94.24
		1 (Δ3)	D	3	0.075	0.618	61.76	90.50
		2 (Δ3)	E	1	0.040	0.648	64.78	94.94

The position of amino acid residues in the epitope was based on the Pan Y et al., 2016.
Δ2 defined as the circulating strain with 162-163 deletions in HA1 sequence and only compared with B/Colorado/06/2017.
Δ3 indicated the circulating strain with 162-164 deletions in HA1 sequence and only compared with B/Washington/02/2019.
E (68.24%) was calculated from $E = -0.864 \times P_{epitope} + 0.6824$ in which efficacy is 68.24% when the $P_{epitope} = 0$.
E (100%) = E (68.24%) *100 /68.24.

A)

A(H1N1)pdm09	HA1																HA2			
									RBS	RBS	RBS	RBS								
Antigenic sites	Cb				Sa	Sa	Sa		Sb	Sb	Sb									
Amino aciid position	74	120	129	130	156	161	164	183	185	187	189	223	235	250	260	271	295	77	124	179	193
**A/Michigan/45/2015 (6B.1)**	S	T	N	K	N	L	S	S	T	D	Q	Q	D	V	N	P	I	I	N	E	A
6B.1	.	.	.	.	.	.	.	.	.	.	.	Q/R	.	.	.	P/Q	.	.	.	.	.
6B.1A	R	.	.	.	.	.	T	.	.	.	.	.	.	.	.	.	V	M	.	.	.
6B.1A1	R	.	.	.	.	.	T	P	T/I	.	.	.	.	.	.	.	V	M	N/T	.	.
6B.1A5	R	.	.	.	.	.	T	P	.	.	.	.	.	.	D	.	V	M	.	.	.
6B.1A5A	R	.	D	.	.	.	T	P	I	.	.	.	E	.	D	.	V	M	.	.	V
6B.1A5A +N156K	R	.	D	N	K	I	T	P	I	.	.	.	.	A	D	.	V	M	.	D	V
6B.1A5A+D187A+Q189E	R	.	D	.	.	.	T	P	I	A	E	.	.	.	D	.	V	M	.	.	V
6B.1A6	R	A	.	.	.	.	T	P	.	.	.	.	.	.	.	.	V	M	.	.	.

B)

A(H3N2)	HA1																				HA2							
								RBS	RBS	RBS		RBS				RBS												
Antigenic sites	E	E	E	D	D	B	A	A	A	A	A	A	B	D	B	B	D	D	E	C								
Amino aciid position	62	91	92	96	121	128	131	135	137	138	142	144	160	171	193	194	212	246	261	311	17	77	149	150	155	160	200	201
**A/Hong Kong/4801/2014 (3C.2a)**	E	S	K	N	N	T	T	T	S	A	R	S	T	N	F	L	A	N	R	H	M	I	I	E	G	D	V	A
3C.2a1	.	.	.	.	K	.	.	.	.	.	G/K	.	.	K	.	.	.	.	.	.	.	V	.		E	.	.	.
3C.2a1b	G	.	R	.	.	.	.	.	.	.	G	.	.	K	.	.	.	.	.	Q	.	.	.	G	E	.	.	.
3C.2a1b+T131K	G	.	R	.	.	.	K	.	.	.	G	.	.	K	.	.	.	.	.	Q	.	.	.	G	E	.	I	.
3C.2a1b+T135K	G	.	R	.	.	.	.	K	.	.	G	.	.	K	.	.	.	.	.	Q	.	.	.	G	E	.	.	.
3C.2a1b+T135K-A	G	.	R	.	.	A	.	K	.	.	G	.	.	K	.	.	.	.	.	Q	.	.	.	G	E	.	.	.
3C.2a1b+T135K-B	G	.	R	.	.	A	.	K	F	S	G	.	.	K	S	.	.	.	.	Q	.	.	.	G	E	.	.	.
3C.2a2	.	.	.	S	.	.	K	.	.	.	K	.	K	.	.	P	T	.	Q	.	.	.	.	.	.	.	.	.
**A/Switzerland/9715293/2013 (3C.3a)**	E	S	K	N	N	A	T	T	S	S	G	N	K	N	F	L	A	N	R	Q	M	I	I	G	G	D	V	A
3C.3a	.	N	.	.	.	.	.	.	.	.	.	K	.	.	S	.	.	E	.		L	.	M	.	.	N	.	V

C)

B/Yamagata	HA1	
Antigenic sites		
Amino aciid position	172	251
**B/Phuket/3073/2013 (Yam 3)**	L	M
Yamagata 3	Q	V

D)

B/Victoria	HA1								
Antigenic sites	Adj 120 loop	120 loop		Adj 120 loop					190 helix
Amino aciid position	117	129	133	136	162	163	164	180	197
**B/Brisbane/60/2008 (Vic 1A)**	I	N	G	K	K	N	D	I	D
VIC1A	V	D	.	.	.	.	.	.	N
vic1A del2	V	D	.	.	-	-	.	V	N
Vic1A del3-A	V	D	.	.	-	-	-	T	N
Vic1A del3-B	V	D	R	E	-	-	-	.	N

Supplementary Figure 1. Sequences Alignment of individual clade/subclade was found in Thai strains. Residue changes between vaccine strains (shown in bold) and the subclade of Thai circulating strains were observed in A) A(H1N1)pdm09 by compared with A/Michigan/45/2015 (accession no. EPI662594; passage history: MDCK1), B) A(H3N2) by compared with A/Hong Kong/4801/2014 (accession no. EPI539576; passage history: SIAT1), C) B/Yamagata-like lineage by compared with B/Phuket/3073/2013 (accession no. EPI529345; passage history: MDCK1) and D) B/Victoria-like lineage by compared with B/Brisbane/60/2008 (accession no. EPI366461; passage history: eggs). Adj defined as AA position located adjacent antigenic site.

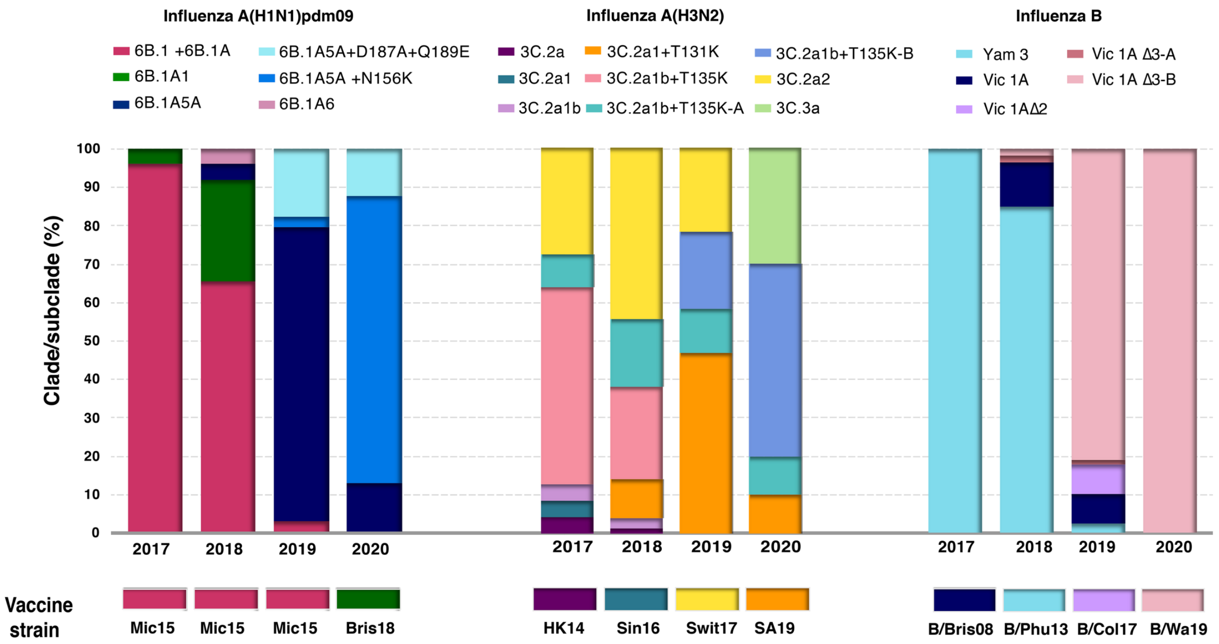

Supplementary Figure 2. Distribution of clades/subclades of circulating influenza viruses in Thailand and their vaccine strain. The bar graph was generated by using Microsoft Excel (Office 365).

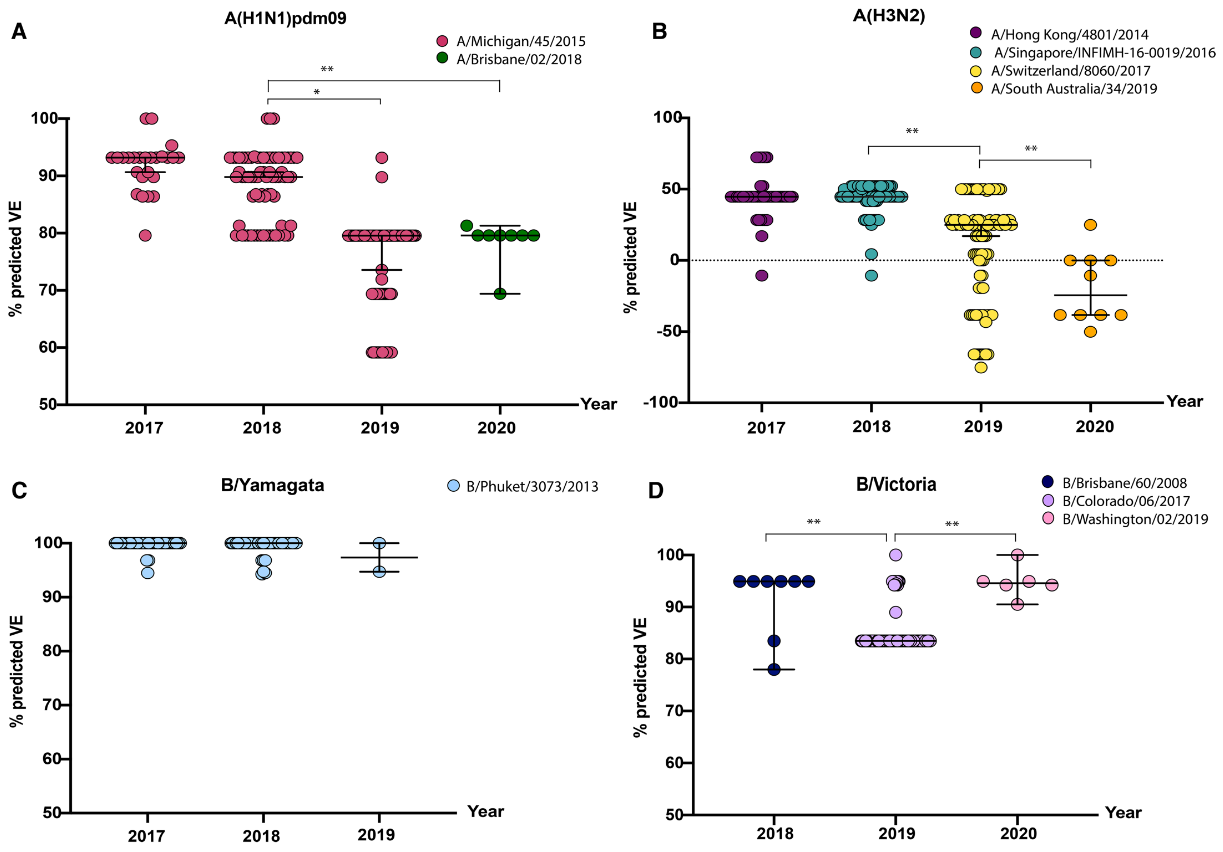

Supplementary Figure 3. Comparison of the yearly computed antigenic relatedness based on HA1 sequences between July 2017 and March 2020. Colored circles indicate the computed antigenic relatedness for A(H1N1)pdm09 (A), A(H3N2) (B), B/Yamagata-like lineage (C), and B/Victoria-like lineage (D) compared with their vaccine. For each year, the middle bar represents the median value with 95%CI. P-values were calculated using one-way ANOVA. *$p < 0.01$, **$p < 0.001$. All data were presented using GraphPad Prism version 8.0 (GraphPad, San Diego, CA; https://www.graphpad.com)
